# Does smoking among friends explain apparent genetic effects on current smoking in adolescence and young adulthood?

**DOI:** 10.1038/sj.bjc.6604250

**Published:** 2008-03-04

**Authors:** V M White, G B Byrnes, B Webster, J L Hopper

**Affiliations:** 1 The Centre for Behavioural Research in Cancer, The Cancer Council Victoria, Carlton, Victoria 3053, Australia; 2 The Centre for Molecular, Environmental, Genetic and Analytic Epidemiology, School of Population Health, The University of Melbourne, Victoria 3010, Australia

**Keywords:** smoking, adolescence, young adults, twins, longitudinal

## Abstract

We used data from a prospective cohort study of twins to investigate the influence of unmeasured genetic and measured and unmeasured environmental factors on the smoking behaviour of adolescents and young adults. Twins were surveyed in 1988 (aged 11–18 years), 1991, 1996 and 2004 with data from 1409, 1121, 732 and 758 pairs analysed from each survey wave, respectively. Questionnaires assessed the smoking behaviour of twins and the perceived smoking behaviour of friends and parents. Using a novel logistic regression analysis, we simultaneously modelled individual risk and excess concordance for current smoking as a function of zygosity, survey wave, parental smoking and peer smoking. Being concordant for having peers who smoked was a predictor of concordance for current smoking (*P*<0.001). After adjusting for peer smoking, monozygotic (MZ) pairs were no more alike than dizygotic pairs for current smoking at waves 2, 3 and 4. Genetic explanations are not needed to explain the greater concordance for current smoking among adult MZ pairs. However, if they are invoked, the role of genes may be due to indirect effects acting through the social environment. Smoking prevention efforts may benefit more by targeting social factors than attempting to identify genetic factors associated with smoking.

Smoking is an important cause of preventable mortality and morbidity in later life (Ezzati and Lopez, 2004), so there is a need to understand the factors associated with its uptake and establishment. Twin studies have the potential to identify whether genetic factors might play a role in explaining individual variation in smoking behaviours. The observation that identical (monozygotic; MZ) twin pairs are more similar than same-sex non-identical (dizygotic; DZ) twin pairs is often interpreted as showing that genetic factors play a role, because this finding is consistent with such an explanation under the assumptions of the classic twin model (CTM). One of the main assumptions of the CTM is that the effects of the shared environment on the relevant trait are the same for MZ and DZ pairs (the equal environments assumption (EEA)). Under this assumption, any greater similarity for MZ pairs compared with DZ pairs is attributed to their greater genetic similarity.

Using the CTM, studies of the smoking behaviour of adult twins have been interpreted as showing that genetic factors play a major role in both initiation and persistence of smoking (Carmelli *et al*, 1992; Heath and Martin, 1993; Heath *et al*, 1993, 1999; Madden *et al*, 1999, 2004; Maes *et al*, 2006). Environmental and lifestyle factors shared by twins have been found to play only a small role in adult smoking (Sullivan and Kendler, 1999; Li *et al*, 2003), although they may be more important in the smoking behaviours of adolescents and young adults (Boomsma *et al*, 1994; Han *et al*, 1999; Koopmans *et al*, 1999; McGue *et al*, 2000; Hopfer *et al*, 2003; Rhee *et al*, 2003; White *et al*, 2003).

There is increasing recognition that violations of the EEA for smoking may influence heritability estimates for smoking and therefore its adequacy needs examination (Kendler and Gardner, 1998; Rende *et al*, 2005; Pergadia *et al*, 2006; Prescott *et al*, 2006; Kaprio, 2007; Tishler and Carey, 2007). Several genetically informative sibling studies have found that the role of the common environment in explaining variation in smoking is greater among siblings (including twins) who share friends than those who do not (Madden *et al*, 2004; Rende *et al*, 2005). Although various interpretations have been given for these findings, there is some agreement that considering social influences on smoking in genetically informative designs may increase our understanding of the aetiology of smoking (Conger, 2005; Merikangas, 2005; Rende *et al*, 2005; Dick *et al*, 2007). We have conducted a prospective, longitudinal study of smoking from adolescence to adulthood using MZ and DZ twin pairs, utilising a social influence framework to understand smoking uptake. Twins were measured at four times (waves 1–4) spanning 17 years, with the median age of the twins increasing from 15 years at wave 1 to 31 years at wave 4. During this time, the twins began to live apart and spend substantially less time with one another. Data on the use of tobacco as well as information on the smoking behaviours of friends and parents, factors found to be influential in adolescent smoking behaviour (Conrad *et al*, 1992; Tyas and Pederson, 1998), were collected at each wave.

We used data from this study to investigate more fully the relative influence of friends' smoking, genes and other non-genetic factors on smoking during adolescence and young adulthood. We also used a novel analytic method that allows us to study factors that may modify both individual behaviours and the similarity of behaviour within twin pairs. We focus on current smoking, rather than whether participants had ever smoked, as it is a person's continued current smoking that is of greatest relevance to their future health.

## MATERIALS AND METHODS

### Procedure

The procedures for recruiting the sample into wave 1 have been described previously (Hopper *et al*, 1992; White *et al*, 2003). In brief, during 1988, questionnaires were mailed to adolescent twins (then aged 9–19 years) registered with the Australian Twin Register via their parents, and completed questionnaires were received from 2863 twins, of whom 1417 were pairs, representing a 97% pairwise response. During 1991, the parents and twins participating at wave 1 were approached by letter and asked to complete the wave 2 survey. At wave 2, 2356 completed questionnaires were returned. Five years later, the wave 3 questionnaire was mailed to all twins participating in the wave 1 survey and a total of 1841 were returned. In 2004, the 2726 twins from wave 1 still registered with the Australian Twin Register were approached and 1884 participated in the study (259 were not contactable and 329 withdrew). Based on the number of contactable twins, a 79% response rate at wave 4 was achieved with 66% of individuals participating at wave 1 also participating at wave 4. At wave 4, 773 twin pairs participated. For this paper, we used data from the pairs who participated at wave 1, were aged 11–18 years (comprising 99% of wave 1 pairs) and who participated in a subsequent wave. Table 1 shows the number of pairs by type at each wave used in these analyses. Twin pair-type predicted the probability of the twins participating in the subsequent survey wave, with same-sex DZ twins (odds ratio (OR)=0.81, *P*=0.025) and opposite-sex DZ twins (OR=0.65, *P*<0.001) being less likely to return than MZ twins. These effects were approximately constant across waves, as indicated by the lack of significant interaction between wave and pair-type.

### Dependent variable: current smoking

At waves 1, 2 and 3, respondents indicating that they had smoked in the week before the survey were defined as current smokers. At wave 4, respondents indicating that they smoked daily or at least weekly were classified as current smokers.

### Smoking behaviours of parents, peers and co-twins

At each wave, respondents indicated the perceived smoking status of their mother, father, co-twin and for each of up to four friends. Respondents classified their parents as being a ‘non-smoker’, an ‘ex-smoker’ or a ‘smoker’. We focussed on current smoking of parents. Due to small numbers reporting that both parents smoked (see Table 1), parental smoking was classified into two groups: neither parent currently smoked or at least one parent currently smoked.

At each wave, twins reported on the smoking status (non-smokers, ex-smokers, occasional, light or heavy smokers) of up to four of their closest friends. The proportion of friends who engaged in any kind of smoking was determined by dividing the number of smoking friends by the total number of friends listed. For correlations and logistic regressions, friends' smoking was classified into two groups: no smokers among friends or at least one smoker.

At each wave, twins were asked if they were living with their twin, as well as the frequency of meeting their twin, with responses classified into every day, at least weekly and less than weekly.

### Statistical analyses

Proportions of individual twins who were current smokers and who had friends who smoked were calculated by wave and pair-type. As there is no *a priori* reason to prefer tetrachoric correlations to other measures, we estimated both the Pearson and tetrachoric twin-pair correlations (*r*) for current smoking status and friends' smoking status for each wave and zygosity group. Under the assumptions of the CTM (see Introduction), we estimated naïve heritability as 2(*r*_MZ_−*r*_DZ_) and examined its dependence on correlation type and wave.

We estimated parameters in a single model to describe both the probability of current smoking for individual twins and the probability of concordance of current smoking for pairs. Pairs were defined as being concordant for their smoking behaviour at a given wave if both were current smokers or if both were not current smokers. Note that specifying the probability of each twin smoking and the probability that the pairs are concordant for smoking is equivalent to specifying the probabilities of each of the four possible pair-smoking states: both smoke, two combinations where only one smokes and neither smoke (see Appendix).

We used logistic regression to model both individual smoking and pair concordance, and estimated the two sets of parameters simultaneously by maximum likelihood. As the probability of an individual smoking is not independent of the probability of pair concordance (e.g., if everyone smokes, all pairs will be concordant), we included a compensating term in the predictor of concordance such that if all coefficients (log-odds for concordance) were zero, the predicted probability of concordance would be exactly as if twins in the same pair were uncorrelated in their smoking behaviours. Hence, in effect, we estimated predictors of excess concordance. To allow for correlation between observations on the same pairs in separate waves, robust (Huber–White) estimates of standard errors were used. The optimisation procedure was coded in Stata 8 and Stata 9 (StataCorp, 2003, 2006), using the ML package. Multiple starting points were used for each run and the progress of the fitting algorithm was monitored to determine, so far as possible, that convergence was to the global maximum likelihood. Convexity of the likelihood surface was also checked using simulated data, by plotting in the neighbourhood of the analytically determined maximum. The order of twins was randomly permuted at the start of each run to avoid the possibility of bias due to any unplanned systematic ordering within pairs. Further details of the method are given in the Appendix. Predictors of individual current smoking could be either individual-specific (sex, smoking among peers) or pair-specific (i.e., zygosity, parental smoking). Predictors of concordance were necessarily pair-specific (zygosity, parental smoking, same or different sex and age). To capture any time dependence of effects, the wave number was included in the initial model, both as a main effect and in interaction with zygosity.

To minimize bias due to unequal duration of participation, only those variables that were measured at each wave or that remained constant with time (e.g., sex) were used in the analysis. Only those observations for which both twins participated at the wave could be retained, due to the pairwise nature of the analytic method.

## RESULTS

### Sample description

Smoking status of the twin pair was associated with subsequent survey participation. Pairs in which one (OR=0.62, *P*<0.001) or both (OR=0.49, *P*<0.001) smoked were less likely to participate in a subsequent survey than were pairs in which neither smoked. However, pairs concordant for smoking status were not significantly more likely to remain in the study (OR=1.12, *P*=0.25) than non-concordant pairs.

The proportion of participating twins who were current smokers increased from wave 1 to wave 3, whereas the proportion of twins with no smokers among their friends decreased (see Table 1). The proportion of twins indicating that neither parent currently smoked also decreased between waves 1 and 3.

**Figure 1:**
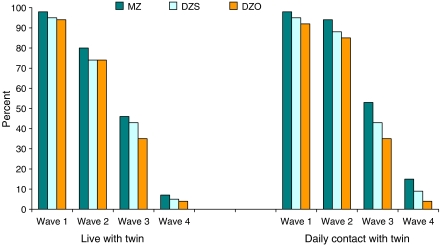
Proportion of twins by zygosity who live with their twin (left) and meet with their twin everyday (right), by survey wave. DZO, opposite-sex dizygotic or fraternal twins; DZS, same-sex dizygotic or fraternal twins; MZ, monozygotic or identical twins.

Figure 1 shows that the proportion of twins cohabiting decreased over the period of the study. The frequency of contact within pairs also decreased for all twin pair-types as the twins aged. There was an association between zygosity and contact, with more MZ twins reporting daily contact with their co-twin than DZ twins.

Table 2 shows that, at each wave, the prevalence of current smoking for MZ twins was lower than for DZ twins. At each wave, and regardless of twin pair-type, twins who were current smokers were much more likely to have smokers among their friends than twins who were not current smokers: about 90% of twins who smoked had smokers among their friends at each survey wave.

### Correlations

Table 3 shows that for current smoking, the tetrachoric correlations were in general higher than Pearson correlations, which translated into higher naïve heritability estimates at waves 2, 3 and 4. There appeared to be a decline in naïve heritability defined in these terms between waves 3 and 4, more so when measured by Pearson correlation.

Table 3 also shows the Pearson and tetrachoric correlations for friends' smoking status and the naïve heritability estimate for each wave. Correlations were higher for MZ pairs than for DZ pairs and again the tetrachoric correlations were higher than the Pearson correlations.

### Associations with smoking and concordance

Table 4 shows the estimates of multivariate ORs for both the probability of individuals smoking and the probability of excess concordance of current smoking under two models. Model 1 includes zygosity, parental smoking and wave as predictors of individual smoking. For excess concordance, it includes zygosity and its interaction with wave (shown in Table 4 as the effect of zygosity at each wave). All estimates shown are adjusted for other variables in the model. Overall, MZ twins were less likely to smoke (*P*<0.001), and this association was consistent across all waves (there was no interaction with wave number). There was a significant effect of wave indicating the greater probability of being a current smoker with increasing age. Regarding pair concordance, MZ pairs were more alike than DZ pairs for their smoking behaviours at waves 1, 2 and 3 (*P*=0.003, 0.004 and 0.03, respectively).

Model 2 adds to Model 1 the effect of friends' smoking at the individual level as well as the concordance of friends' smoking at the pair level. There was a strong association between an individual's smoking status and that of their peers (OR=10.9, *P*<0.001), which was far greater than the association with parental smoking (OR=1.75, *P*<0.001). There was no evidence that the parental or peer associations varied by wave.

When modelling the probability of pair concordance for current smoking, concordance of friends' smoking status was a significant predictor and this association did not differ significantly across waves. Including concordance for friends' smoking in Model 2 reduced the difference between MZ and DZ concordance for smoking estimated under Model 1 at waves 2, 3 and 4, such that the effect of zygosity was no longer significant at these waves.

## DISCUSSION

Consistent with most other investigators, we found greater correlation for current smoking in MZ twin pairs than in DZ twin pairs using both Pearson and tetrachoric correlation estimates. The corresponding heritability estimates under the assumptions of the CTM were consistent with values reported from various twin studies (Carmelli *et al*, 1992; Heath and Martin, 1993; Heath *et al*, 1993, 1999; Boomsma *et al*, 1994; Han *et al*, 1999; Koopmans *et al*, 1999; Madden *et al*, 1999, 2004; McGue *et al*, 2000; Rhee *et al*, 2003; Maes *et al*, 2006). However, using a novel analysis, which allowed us to adjust for both the smoking status of an individual's friends and the concordance for friends' smoking status within pairs, we found greater concordance for smoking in MZ pairs only at wave 1 when most twins were living together.

There is considerable evidence in the literature that the smoking behaviour of friends has a major influence on the current and future smoking of adolescents and young adults (Flay *et al*, 1994, 1998; Distefan *et al*, 1998; Engels *et al*, 1999; Chassin *et al*, 2000; Leatherdale *et al*, 2005). If, as our study found, MZ pairs are more similar in their friends' smoking behaviours than are DZ pairs, this could explain some or all of the greater correlation of smoking observed in MZ twins. Our study found this to be the case, at least during the time when twins start to live apart.

If MZ pairs share a more similar environment than DZ pairs, then either the EEA of the CTM is invalid, or the environment must be considered a manifestation of the twins' genes. The first possibility would result in biased heritability estimates; the second would imply a broad notion of heritability, part of which may be subject to environmental modification. This is the standard interpretation of heritability used in zoology, where it is used to determine the response to selection (Mulder *et al*, 2007). The EEA has been tested only under a limited number of circumstances (Loehlin, 1992) and for only some substances, and the findings have been mixed (Prescott *et al*, 2006). Although we did not formally assess this assumption, we found that, compared with DZ twins, MZ twins had more frequent contact with each other at all survey waves and their friends were more similar in smoking behaviours. Other work has also found that adolescent MZ twin pairs spend more time together and share more friends than do DZ twin pairs (Rende *et al*, 2005), and that MZ pairs are more dependent on their co-twin than DZ pairs (Penninkilampi-Kerola *et al*, 2005). These findings are in line with the suggestion that a ‘special MZ environment’ might contribute to the greater similarities in the smoking behaviours of MZ twin pairs (Stallings *et al*, 1999). Directly measuring and adjusting for differences in shared environment may help reduce any resultant bias on heritability estimates. Several recent studies involving adolescents have commenced this investigation (Rende *et al*, 2005; Slomkowski *et al*, 2005; Pergadia *et al*, 2006; Dick *et al*, 2007). Dick *et al* (2007) found that adjusting for parental contact influenced heritability estimates for smoking and suggested that different environments moderate genetic effects on the variability in tobacco use. Rende and colleagues found that adjusting for shared friends and amount of contact between twins influenced the role of the shared environment, but not genes, on smoking variability, suggesting to them a sibling ‘contagion effect’ that operates through environmental processes (Rende *et al*, 2005; Slomkowski *et al*, 2005). None of these studies modelled the influence of concordance of friends' smoking in their models.

To disentangle the possible confounding of the effects of genes and friends, we developed an analytic method that could adjust for measured covariates of smoking. Our approach allowed us to include both the smoking status of each twin's friends and the concordance of friends' smoking status of a twin pair. After adjusting for these factors, there was no evidence for increased concordance in MZ pairs at waves 2, 3 or 4.

A necessary consequence of a genetic contribution to behaviour is that MZ pairs are more alike in that behaviour than are DZ pairs. If this difference is not observed then it is problematic to accept the hypothesis of a genetic contribution. A more difficult question is whether greater similarity in MZ pairs is sufficient to conclude a role for genes. Our findings suggest that it is possible to explain the greater concordance in smoking for MZ pairs compared with DZ pairs at waves 2–4 without reference to unmeasured genetic factors. Monozygotic pairs may be more alike in their smoking for the simple reason that their friends are more alike in their smoking.

Two outstanding issues remain: what is the source of greater concordance for smoking in MZ pairs at wave 1; and what is the source of greater concordance for the smoking status of the friends for MZ pairs at all waves.

Regarding the first issue, although the pattern of results could suggest genetic factors influencing smoking ‘turn-on’ during adolescence and ‘turn-off’ during young adulthood, it may also suggest that there is unmeasured confounding due to greater shared environment for MZ pairs while living together.

For the second issue, it is possible that the greater social contact or connectedness between MZ twins than DZ twins (Penninkilampi-Kerola *et al*, 2005; Rende *et al*, 2005) simply leads to twins sharing more friends. However, as the literature based on the CTM provide some evidence that the choice of friends (Baker and Daniels, 1990; Iervolino *et al*, 2002) and exposure to friends who smoke (Cleveland *et al*, 2005) are influenced by genetic factors, it is also possible to suggest that the influence of friends on smoking behaviour is, at root, genetic. From this position and assuming that the EEA is correct, our findings could suggest that genes influence the smoking behaviours of adolescents and young adults indirectly by influencing friendship selection. One motivation for seeking genes influencing smoking behaviour is that their discovery could provide a target for pharmaceutical interventions, by either blocking or enhancing the action of the proteins encoded by the genes (Tyndale, 2003). However, a gene that modifies smoking indirectly by influencing the selection of friends would be a less likely target for pharmaceutical interventions. If genetic explanations of smoking are to be made, research needs to distinguish the contribution of direct and indirect genetic effects, as this will determine whether searching for specific genes associated with smoking is likely to be fruitful. If genetic effects on smoking mainly act through environmental mechanisms as is suggested by our results, then social interventions may be the most effective means at reducing smoking.

Although this study has a number of strengths, including its longitudinal nature and assessing friends' smoking status at each survey wave, several limitations need to be kept in mind. First, there was attrition from waves 1 to 4. Smokers were less likely to participate in the study at later waves than non-smokers, as were DZ twins. This could lead to a false association between zygosity and individual smoking status, with the progressive concentration with each wave expected to produce an increasingly strong association between zygosity and smoking. However, this was not observed. Second, we studied current smoking status at each wave rather than studying the status of ever having been a current or regular smoker or ever smoking, as has been usual in the behavioural genetics smoking literature. We adopted this strategy due to the young age of our sample at the first wave. The notion of an adolescent as a smoker is more ambiguous than it is among adults, with research suggesting that a non-trivial proportion of older adolescents who describe themselves as a smoker refer to themselves as a ‘non-smoker’ 6 months later (Schofield *et al*, 1998). Finally, smoking was more common among our DZ twins than MZ twins at all survey waves. Although this finding must be considered with caution due to differential retention in later waves, as discussed above, it may indicate a violation of the EEA leading to inflated heritability estimates (Tishler and Carey, 2007). Although the veracity of this suggestion is being debated (Kaprio, 2007), increasing concern about the appropriateness of the EEA for smoking led us to describe our heritability estimates as ‘naïve’.

Recently, there has been a call for researchers studying twins to investigate how environmental factors might mediate or influence the putative role of genes (Penninkilampi-Kerola *et al*, 2005). Our study has attempted to do this by using a novel analytic approach that does not rely on the assumptions of the CTM to examine the influence of zygosity and peer smoking on the current smoking behaviours of adolescent twins as they grow to adulthood. Once concordance for peer smoking was adjusted for, MZ pairs were no more likely to be concordant for smoking than DZ pairs in late adolescence and adulthood. Our results suggest that genetic explanations do not need to be invoked to explain the greater concordance for smoking in MZ pairs. Although further research is needed to confirm our findings, we believe that they are in line with results from genetically informative designs showing the importance of the social environment on smoking uptake (Rende *et al*, 2005; Slomkowski *et al*, 2005) and in moderating the influence of genes (Dick *et al*, 2007). This growing body of work provides support for the suggestion that smoking prevention efforts may benefit more by targeting social influences than attempting to identify genes associated with smoking (Merikangas, 2005). This public health approach should include well-funded mass media anti-tobacco advertising campaigns, restrictions on smoking in public, increased prices for cigarettes and removal of all tobacco product advertising (Laugesen *et al*, 2000). Since the late 1980s, Australia has adopted many of these policies and the prevalence of smoking among adolescents aged 12–17 years has fallen from 22% in 1984 to 9% in 2005 (White and Hayman, 2006).

## Figures and Tables

**Table 1:** Characteristics of participants in each survey wave (base: individual twins)

**Characteristic (*n*)**	**Wave 1 (2818)**	**Wave 2 (2242)**	**Wave 3 (1464)**	**Wave 4 (1516)**
*Average age*	14.91	18.00	22.51	30.75
				
*Twin type*				
MZ (pairs)	1310 (655)	1076 (538)	752 (376)	746 (373)
DZS (pairs)	824 (412)	652 (326)	406 (203)	436 (218)
DZO (pairs)	684 (342)	514 (257)	306 (153)	334 (167)
				
*Gender*				
Males (%)	46	44	41	41
Females (%)	54	56	59	59
				
*Smoking status*				
Never smoked (%)	37	23	22	13
Experimenter (%)	49	54	49	55
Current smoker (%)	14	23	29	20
Ex-smoker (%)	NA	NA	NA	11
				
*Friend's smoking*				
No friends smoke (%)	65	44	39	46
<50% smoking (%)	20	32	42	39
⩾50% smoking (%)	15	25	19	16
				
*Parent's current smoking*				
None (%)	65	69	77	77
One parent (%)	26	24	19	18
Both parents (%)	10	7	4	5
				
*Meet with twin*				
Everyday (%)	96	81	46	11
Weekly (%)	2	10	22	32
Less than weekly (%)	2	10	33	57

DZO=opposite-sex dizygotic or fraternal twins; DZS=same-sex dizygotic or fraternal twins; MZ=monozygotic or identical twins; NA=not applicable.

NB: % may not add to 100 due to rounding.

**Table 2:** For each twin type, percentage of current smokers at each survey wave and percentage with any smokers among their friends by smoking status, at each survey wave (base: individual twins)

	**Twin type**			
	**MZ**	**DZS**	**DZO**	**Total**
*Current smokers*				
Wave 1 (%)	12	14	17	14
Wave 2 (%)	20	25	26	23
Wave 3 (%)	26	31	31	29
Wave 4 (%)	19	21	24	20
				
*Any smokers among friends*				
*Wave 1*				
Non-smokers (%)	24	30	27	26
Current smokers (%)	90	94	84	90
Total (%)	32	39	37	35
				
*Wave 2*				
Non-smokers (%)	46	43	48	46
Current smokers (%)	93	91	91	92
Total (%)	56	54	59	56
				
*Wave 3*				
Non-smokers (%)	51	49	49	50
Current smokers (%)	90	88	86	88
Total (%)	61	61	61	61
				
*Wave 4*				
Non-smokers (%)	44	47	45	45
Current smokers (%)	90	91	92	91
Total (%)	53	56	56	54

DZO=opposite-sex dizygotic or fraternal twins; DZS=same-sex dizygotic or fraternal twins; MZ=monozygotic or identical twins.

**Table 3:** Intraclass correlations (s.e.) for current smoking in twins and for smoking among friends, by zygosity and wave (base: twin pairs)

	**Wave 1**	**Wave 2**	**Wave 3**	**Wave 4**
*Current smoking in twins*				
*Pearson correlation*				
MZ	0.65 (0.05)	0.58 (0.04)	0.52 (0.05)	0.44 (0.06)
DZS	0.35 (0.05)	0.29 (0.04)	0.25 (0.05)	0.24 (0.06)
2(*r*_MZ_−*r*_DZ_)^a^	0.61 (0.13)	0.59 (0.12)	0.53 (0.14)	0.39 (0.17)
				
*Tetrachoric correlation*				
MZ	0.89 (0.03)	0.82 (0.04)	0.75 (0.05)	0.69 (0.06)
DZS	0.59 (0.06)	0.47 (0.06)	0.41 (0.08)	0.42 (0.08)
2(*r*_MZ_−*r*_DZ_)^a^	0.61 (0.06)	0.71 (0.07)	0.68 (0.09)	0.55 (0.10)
				
*Smoking among friends*				
*Pearson correlation*				
MZ	0.58 (0.04)	0.45 (0.04)	0.38 (0.04)	0.39 (0.06)
DZS	0.42 (0.03)	0.33 (0.04)	0.19 (0.06)	0.11 (0.05)
2(*r*_MZ_−*r*_DZ_)^a^	0.33 (0.10)	0.23 (0.11)	0.38 (0.14)	0.57 (0.16)
				
*Tetrachoric correlation*				
MZ	0.80 (0.03)	0.65 (0.05)	0.57 (0.07)	0.58 (0.06)
DZS	0.62 (0.04)	0.50 (0.06)	0.30 (0.08)	0.42 (0.08)
2(*r*_MZ_−*r*_DZ_)^a^	0.37 (0.05)	0.30 (0.07)	0.54 (0.11)	0.32 (0.10)

DZS=same-sex dizygotic or fraternal twins; MZ=monozygotic or identical twins.

a 2(*r*_MZ_−*r*_DZ_)=provides naive heritability estimates.

**Table 4:** Multivariate ORs jointly estimated for individual current smoking and excess concordance of smoking within twin pairs

	**Model 1**			**Model 2**		
**Factor**	**OR**	**95% CI**	***P*-value**	**OR**	**95% CI**	***P*-value**
*Individual current smoking*						
Monozygosity	0.77	0.63–0.94	0.01	0.77	0.62–0.94	0.01
Parents smoke	1.86	1.63–2.11	<0.001	1.75	1.45–2.09	<0.001
Wave 2	2.72	2.29–3.22	<0.001	1.20	0.99–1.46	0.06
Wave 3	1.70	1.42–2.04	<0.001	1.61	1.29–2.02	<0.001
Wave 4	1.93	1.64–2.27	<0.001	1.08	0.87–1.34	0.5
Friends' smoking	NA			10.90	7.90–15.10	<0.001
						
*Excess concordance of current smoking*						
Monozygosity (wave 1)	1.99	1.26–3.15	0.003	2.60	1.47–4.60	0.001
Monozygosity (wave 2)	1.83	1.22–2.76	0.004	1.12	0.78–1.60	0.5
Monozygosity (wave 3)	1.68	1.07–2.66	0.03	1.13	0.79–1.62	0.5
Monozygosity (wave 4)	1.30	0.86–1.98	0.2	1.18	0.66–1.68	0.5
Concordance of friends' smoking	NA			1.55	1.30–1.85	<0.001

CI=confidence interval; OR=odds ratio; NA=not applicable.

Excess concordance also adjusted for main effect of wave (no significant association).

Estimates shown adjusted for all variables in model.

## APPENDIX

We demonstrate below that the joint distribution of smoking (or other binary features) within a pair of twins is equivalently captured by the marginal probability of smoking, together with the probability that the pair are concordant. We also provide further details on the combined regression model we used to estimate the odds ratios of marginal smoking and concordance with respect to various predictors.

As the twins within a pair are assumed to be exchangeable, the joint distribution of a pair is described by the probabilities that both, one or none of the twins in the pair smoke. We denote these by *p*_2_, *p*_1_ and *p*_0_=1−*p*_2_−*p*_1_, respectively.

Now consider the marginal probability *p*_m_ that a twin smokes, which is the probability that a twin chosen at random from the population is a smoker. This random draw can be conducted by first choosing a pair, and then selecting one of the twins in the pair at random. It is easy to see that *p*_m_=*p*_2_+0.5*p*_1_. Similarly, the probability that a pair of twins is concordant is just *p*_c_=*p*_0_+*p*_2_=1−*p*_1_. This relationship can be inverted to yield *p*_2_=*p*_m_+0.5(*p*_c_−1) and *p*_1_=1−*p*_c_, establishing that they are equivalent representations.

It follows that if we have a model for *p*_m_ and another for *p*_c_, then we have a model for the observed joint distribution. We chose to use logistic models for both *p*_m_ and *p*_c_ largely to avoid confusion with the often-assumed probit model, but any generalised linear model for a binary outcome would suffice.

The remaining problem is to disentangle the effect of the marginal probability of smoking from the probability of concordance. Suppose that each twin made an independent decision to smoke, with probability *p*. Then *p*_2_=*p*^2^ and *p*_1_=2*p*(1−*p*), so that *p*_c_=1−2*p*+2*p*^2^. This increases to 1 as *p* approaches either 0 or 1, as would be expected. To compensate for this, we write the generalised linear models in the form

Here *g*_m_ and *g*_c_ are the link functions for the marginal and concordance models, whereas *X*_m_ and *X*_c_ are the corresponding covariates.

Hence, in the null model where *β*_c_=0, the probability of concordance is as it would be if the smoking status of twins was independent.
